# What can we learn from treatments of oral lichen planus?

**DOI:** 10.3389/fcimb.2024.1279220

**Published:** 2024-02-15

**Authors:** Tingting Wu, Yang Bai, Yin Jing, Fangchun Chen

**Affiliations:** ^1^ Stomatological Hospital of Chongqing Medical University, Chongqing, China; ^2^ Chongqing Key Laboratory of Oral Diseases and Biomedical Sciences, Chongqing, China; ^3^ Chongqing Municipal Key Laboratory of Oral Biomedical Engineering of Higher Education, Chongqing, China

**Keywords:** oral lichen planus, erosive type, pharmacological treatment, nonpharmacological treatment, microbes

## Abstract

Oral lichen planus (OLP), a T-lymphocyte-mediated disease of the oral mucosa, has a complex pathogenesis that involves a number of factors. The disease is characterized by recurrent episodes and requires continuous follow up, and there is no curative treatment available. Erosive lichen planus, among others, has a risk of malignant transformation and requires standardized treatment to control its progression. Different clinical subtypes of oral lichen planus require appropriate treatment. Pharmacological treatments are the most widely available and have the greatest variety of options and a number of novel pharmacological treatments are presented as highlights, including JAK enzyme inhibitors. The second is photodynamic therapy, which is the leading physiological treatment. In addition, periodontal treatment and psychological treatment should not be neglected. In this review, we briefly discuss the most recent developments in therapies for oral lichen planus after summarizing the most widely used clinical treatments, aiming to provide different proposals for future clinical treatment.

## 1 Introduction

**Figure 1:**
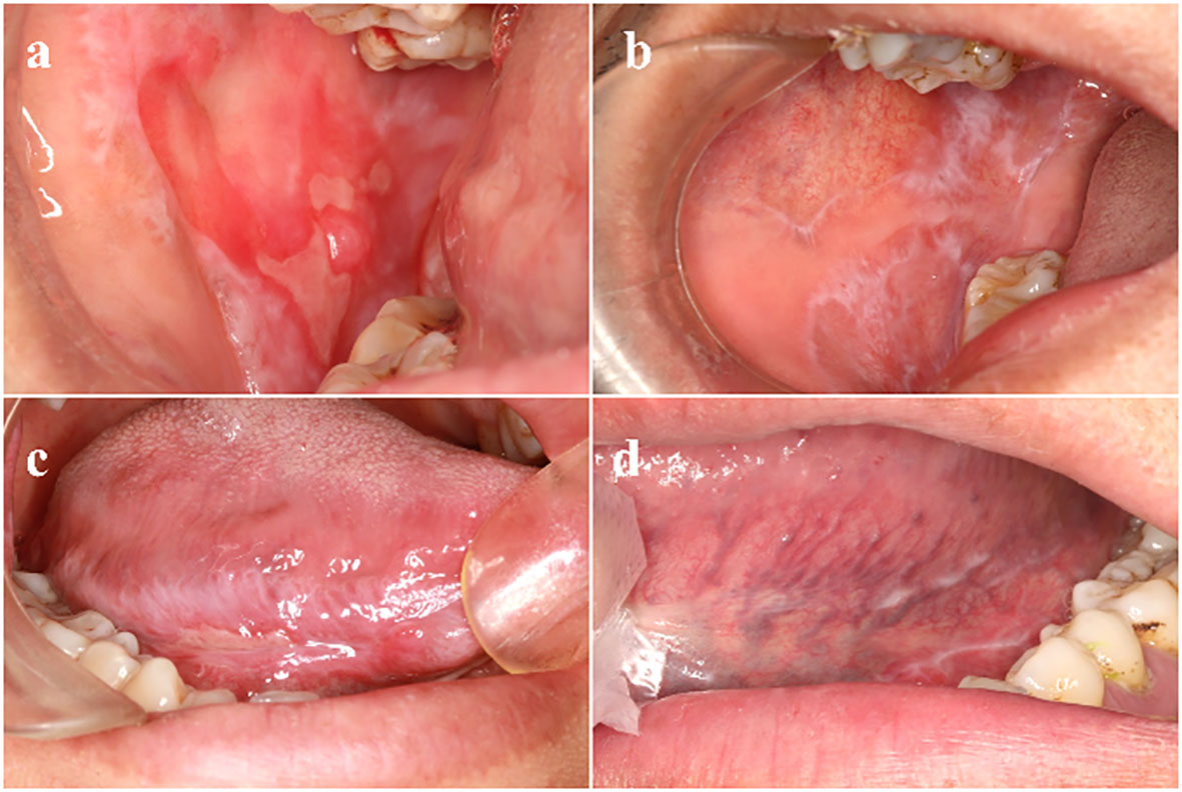
Clinical manifestations of different types of OLP **(A)** Right buccal mucosa: reticular pattern affecting the mucosa, a large erosive area and atrophy seen in the center. **(B)** The white patches and a reticular pattern called Wickham’s striae. **(C)** Right tongue margin: erosive type. **(D)** Left tongue margin: non-erosive type. OLP, oral lichen planus.

Erasmus Wilson first identified lichen planus (LP) in 1869 (Saeed et al., 2022), and oral lichen planus (OLP) is the name for the lesions that develop in the oral mucosa, a chronic inflammatory condition with an autoimmune component and an unknown cause (Carrozzo et al., 2019). OLP is approximately 0.98 percent prevalent worldwide, and the majority of patients are middle-aged women (Li et al., 2020). OLP lesions tend to occur on the buccal mucosa, ventral, and dorsal parts of the tongue’s mucous membranes (**Figure 1**). The typical clinical feature is a bilateral white or reticulated pattern, known as the Wickham striations. Clinically, OLP can be categorized as erosive or non-erosive (Didona and Hertl, 2022). The disorder is presently categorized by the World Health Organization (WHO) as an oral potentially malignant disease due to the possibility of its malignant transformation, with an erosive or ulcerative form considered a high-risk factor (Giuliani et al., 2019). To date, the potential for OLP to become malignant remains a highly controversial issue, with incidence rates fluctuating between 0 and 3.5% (Fitzpatrick et al., 2014), and most authors emphasize that clinical treatment should focus on symptom-based forms of atrophic and erosive OLP (EOLP) (Eisen et al., 2005). As a result, EOLP has become challenging to manage the lesions, prevent recurrence, and minimize side effects complications. There is currently no cure for OLP, and the standard first-line treatment consists of topical corticosteroids (Society of Oral Medicine and Chinese Stomatological Association, 2022). Klieb et al. proposed that the management of OLP can be effectively addressed through a multidisciplinary approach. For instance, patients with OLP who complain of dysphagia need the assistance of a gastroenterologist to rule out esophageal lichen planus (Shavit et al., 2020). The correlation between oral lichen planus (OLP), diabetes mellitus (DM), and hypertension was initially documented in 1990, indicating that OLP should not be regarded solely as a condition confined to the oral mucosa. Hence, it is imperative to conduct a meticulous examination of the patient’s medical history to identify any concurrent comorbidities (Lamey et al., 1990). Secondly, anxiety and psychological depression are risk factors for OLP that should not be ignored, and the onset of OLP itself leads to sleep disturbances and mood disorders, which should be closely monitored and psychologically counseled accordingly (Adamo et al., 2015). This review summarize the existing and latest advancements in OLP treatments to provide ideas for future clinical treatment.

## 2 Pharmacological treatment

The major therapeutic agents for EOLP include corticosteroids, calcineurin inhibitors (cyclosporin-A, tacrolimus), hydroxychloroquine, and total glucosides of paeony, etc. Each type of drug will be discussed in turn below.

### 2.1 Corticosteroids

In development, metabolism, and immunity, adrenal steroid hormones (known as glucocorticoids) can have an important impact, and corticosteroids (CS), which are synthetic analogs of glucocorticoids, have anti-inflammatory and immunosuppressive effects (Ayyar and Jusko, 2020). In their comparison of the costs and effectiveness of several OLP treatment interventions, Sandhu et al. concluded that topical steroids continue to be the most economical and effective choice at the moment (Sandhu et al., 2022). In clinical practice, the most frequently employed agents are triamcinolone acetonide, betamethasone, clobetasol, and dexamethasone, and for systemic application, prednisone (Alrashdan et al., 2016b). *In vivo*, CS inhibits the maturation, differentiation, and proliferation of virtually all immune cells and reduces the inflammatory response by inducing vasoconstriction and reducing blood flow to the lesion (Claman, 1975). Topical application and intra- and submucosal injections of steroids are appropriate for mild to moderate lesions (Serafini et al., 2023). Systemic medications are indicated for recalcitrant and severe multifocal lesions, such as large erosive lesions, in which conventional medications have failed. However, CS have limitations, with the most prevalent side effect being opportunistic fungal infections. The reason for this is because corticosteroids’ immunosuppressive effects, can disturb the oral microbiome rendering the mouth susceptible to a variety of bacterial, parasitic, and viral infections (Hodgens and Sharman, 2023). As a result, CS are frequently used clinically in combination with antifungal drugs such as miconazole or chlorhexidine. On the other hand, CS topical therapy appears to affect the microbiota in OLP in some way. For example, following four weeks of using mouthwash containing 0.05% dexamethasone, Jeong et al. observed alterations in the composition of the microbial population in the majority of OLP patients (Ku et al., 2021), and this could be among the risk factors that cause fungal infections.

EOLP has been successfully treated with clobetasol patches (Rivelin-CLO) in clinical trials recently. This is a patch named Rivelin-CLO that adheres to mucosal surfaces and offers targeted delivery, dose control, and ease of continuous administration compared to traditional forms (Brennan et al., 2022). The innovative regimen was more effective, comfortable, and easy to apply, improving patient compliance. In addition, no adverse effects such as Candida infection were observed during the observation period, suggesting a high safety profile of the drug. However, topical clobetasol propionate gel has been linked to hypoadrenocorticism (Einarsdottir et al., 2023), and the adhesion ability of the patch is affected by the site of the lesion and oral activity, which may cause lingual border and alveolar mucosa patch dislodgement. In another lab study, CS was delivered to a human keratinocyte-T cell co-culture model using a new electrostatic spun mucosal adhesion patch, reducing activated Jurkat T cell IL-2 release. This medication delivery method also enhances CS action in confined lesions (Said et al., 2021). This mode of administration has fewer side effects and adverse reactions, making it a safe first-line therapy for the treatment of EOLP (**Table 1**).

**Table 1:** Corticosteroids in oral lichen planus.

Drug	Mechanism of Action	Indications	Method and Dose	Side Effect
Triamcinolone acetonide	1.Aanti-cell proliferlation 2.Causes eosinophils and lymphocytes to trigger programmed cell death or apoptosis; reduces neutrophil apoptosis 3.Induces vasoconstriction and reduce blood flow to the lesion 4.Usually inhibits the maturation, differentiation and proliferation of all immune cells	**Topical application:** non-erosive/mild-to-moderate erosive OLP patients **Subcutaneous injection:** unresponsive or poorly responsive to topical medications	**Topical application:** Balm: topical application:0.1% TA paste, tid (gums and palate) Suspension(liquid):1mg/ml, t.i.d (Suitable for coated mucous membranes) **Subcutaneous injection:** Topical injection: 0.2-0.4ml(10mg/ml) TA once a week, 2-3 weeks	1.Dysbiosis 2.Systemic complications
Betamethasone		Erosive oral lichen planus topical application	500 mg betamethasone tablets dissolved in 10-15 ml of water for mouthwash, 4 times daily.	
Dexamethasone		OLP with extensive intraoral lesions topical application	0.1mg/ml, 5ml, 3 times/day	
Clobetasol propionate		Erosive oral lichen planus topical application	Clobetasol ointment(0.05%), 3-4 times/day	
Prednisolone		**Systemic administration:** for severe multifocal lesions with large erosions	**Systemic administration: **Adult dose of 40 mg daily (first 5 days), 10-20 mg daily (6-10 days)	

### 2.2 Calcineurin inhibitors

Calcineurin inhibitors are immunomodulators that combine with lecithin in T lymphocytes (cyclosporine binds to cyclophilin; tacrolimus and pimecrolimus bind to the FK506 binding protein), and these medications are used in the management of immune-mediated lesions (Tobón-Arroyave et al., 2004). Calcium phosphatase inhibitors inhibit the expression of pro-inflammatory factors by binding to macrophillin-12 and dephosphorylating NFAT (Yang et al., 2016) (**Table 2**).

**Table 2:** Calcineurin inhibitors in oral lichen planus.

Drug	Mechanism of Action	Indications	Method and Dose	Side Effect
Tacrolimus	1. Binding to FK506 binding protein (FKBP); 2. Inhibits calcium-regulated phosphatase (calmodulin-dependent phosphatase(protein phosphatase 3)(PPP3C now,PP2B before); 3. Suppression of T-lymphocyte activation by down-regulation of interleukin 2 (IL-2) transcription; 4. Reduced reactivity of T lymphocytes to foreign antigens	For second-line treatment of patients who are insensitive to potent and highly effective topical corticosteroid therapy.	0.03%-0.1% cream, ×2/d	1. Mainly mild to moderate burns, erythema, itching; 2. Partially reported folliculitis, acne, Kaposi's varicella-like rash, herpetic eczema and herpes simplex infections 3. Possible carcinogenic effects
Pimecrolimus	1. Binding to FK506-binding protein (FKBP) and inhibition of calcium-regulated phosphatases dephosphorylates nuclear factor of activated T cells (NFAT) 2. Inhibition of T cell activation and synthesis of Th1 and Th2 based inflammatory cytokines (IL2, IL-4, IL-10, IFN-γ)	For patients with erosive oral lichen planus	1% cream, ×2/d	Burning, tingling or itching sensation in the area
Cyclosporine	1. Binds to cyclophilin 2. Dephosphorylation of nuclear factor of activated T cells (NFAT) and inhibition of IL-2 transcription 3. Inhibition of T-cell activation	Available for both non-erosive/erosive types	Topical : 100mg/ml, × 3/d CsA 1.5%, ×2/d Systemic: not recommended	Local burning sensation, gastrointestinal discomfort; breast pressure; dizziness, itching; swollen lips

#### 2.2.1 Tacrolimus

A 5-year trial found that Tacrolimus inhibited lesion progression and improved subjective complaints (Utz et al., 2022). Another study found that the tacrolimus group showed increased mesenchyme expression of the apoptosis marker caspase-3, which may indicate impaired T-cell viability and reduce local inflammation, explaining its role in OLP immunology (Ibrahim et al., 2023). However, one of the side effects of tacrolimus is that topical application of tacrolimus can release neuropeptides such as substance P to stimulate sensory neurons and produce a transient burning or painful sensation (Riano Arguelles et al., 2006). Topical tacrolimus is a crucial second-line treatment for refractory EOLP, and Calcium phosphate inhibitors may help with refractory erosive OLP if steroids fail. Todd et al. reviewed 13 patients with OLP treated with topical tacrolimus, 11 of whom had a definite treatment effect. Seven of them had no subsequent recurrence (Rozycki et al., 2002). Notably, oral tacrolimus caused intestinal microecological dysregulation by decreasing levels of regenerating islet-derived protein 3 in the ileum and increasing intestinal permeability, which resulted in a significant increase in Bacteroides anomalies and Lactobacillus lactis (Toral et al., 2018). On the other hand, Guo et al. demonstrated that tacrolimus can be metabolized by a variety of intestinal commensal bacteria to a novel metabolite, 9-hydroxytacrolimus, a finding that explains the reduced efficacy of oral tacrolimus (Guo et al., 2019). Furthermore, no microbe-specific investigations on the topical administration of tacrolimus to the oral mucosa have been described.

#### 2.2.2 Cyclosporine A

Cyclosporine A (CsA), an immunosuppressive peptide, is another calcium phosphatase inhibitor that selectively and reversibly suppresses CD4^+^ T cell activation (Guada et al., 2016). CsA also used for the oral treatment of refractory LP (Jungell and Malmstrom, 1996; Wang H. et al., 2016). Farahnaz et al. discovered that cyclosporine and methotrexate were successful in treating 33 cases of resistant EOLP (Fatemi Naeini et al., 2020). However, hypertension, altered renal function, gingival hyperplasia, and skin carcinogenicity are all adverse effects connected to oral administration of CsA (Lanese and Conger, 1993; Rouimi et al., 2018; Didona et al., 2022). Cyclosporine mouthwash is useless because mucosal cytochrome P450 deactivates it (Itin et al., 1992), and its mechanism of action relies on its interaction with systemic T-lymphocytes (Sieg et al., 1995). In terms of microorganisms, CsA has been shown in a rat model to reduce the amount of Enterobacteriaceae and Clostridium clusters I and XIV (Olek et al., 2023).

### 2.3 Hydroxychloroquine

The antimalarial drug hydroxychloroquine (HCQ) has been used to treat autoimmune conditions such as lupus erythematosus and rheumatoid arthritis. It modulates the immune system by stabilizing lysosomal enzymes and reducing the production of inflammatory cytokines (Bhanja et al., 2021). Oral HCQ may be used to treat EOLP 90% of OLP patients who received a 6-month pharmacological treatment for HCQ improved (Eisen, 1993). Yeshurun et al. discovered that 81 percent of 21 EOLP patients improved after three months of HCQ treatment. However, it is important to note that long-term use of hydroxychloroquine can result in irreversible retinal damage, hyperpigmentation, and elevated serum creatinine levels. It has been suggested that patients who have not responded to HCQ treatment for 2-4 months should not continue treatment with hydroxychloroquine to avoid accumulation of doses leading to side effects (Yeshurun et al., 2019) (**Table 3**). HCQ diminished regulatory T cells (Tregs) in the peripheral blood of OLP patients, indicating a potential pharmacological mechanism (Zhu et al., 2014). Unfortunately, this study has not been followed up further, while a number of scholars have reached opposite results regarding the mechanism of action of HCQ on Tregs (An et al., 2017; Sadeghpour et al., 2020; Hu et al., 2022). Therefore, a great number of fundamental studies still need to be conducted to thoroughly investigate the efficacy of HCQ in the therapy of OLP.

**Table 3:** Other immune-related drugs in oral lichen planus.

Drug	Mechanism of Action	Indications	Method and Dose	Side Effect
Hydroxychloroquine	Stabilization of lysosomal membranes and inhibition of prostaglandin synthesis, interference with complement-dependent antigen-antibody responses and inhibition of lymphocyte transformation in vitro	Non-erosive/erosive oral lichen planus population (no eye or heart disease)	200 mg twice daily for six months	Mild gastrointestinal symptoms (nausea, vomiting and diarrhea) and neuromuscular symptoms (headache, myalgia and fatigue)
Azathioprine	Suppression of T-lymphocytes	Severe erosive oral lichen planus or with systemic symptoms	50 mg twice daily orally in3 months	No significant adverse reactions
Sirolimus	Potent immunosuppressant with anti-proliferative and tumor suppressive properties	Patients with recalcitrant oral lichen planus	Topical application of RAPA solution (1mg/ml) twice daily	Tingling or burning sensation
Total glucosides of paeony	TGP consistently reduces the number of Treg and Th1 and inhibits T-cell proliferation	Non-erosive/erosive oral lichen planus population, especially for patients with rheumatoid arthritis or chronic constipation	1200mg per day for 6 months	Occasional diarrhea Doesn't need treatment, goes away on its own

HCQ can exert antibacterial and antiviral effects by alkalinizing the intracellular pH of infected cells and inhibiting the glycosylation of viral proteins (Rolain et al., 2007). Keshavarzi found that HCQ had a direct antifungal effect on both A. fumigatus and A. nidulans (Keshavarzi, 2016). However, for the oral cavity, there are no reports on the inhibition of the growth of Candida albicans in the oral cavity.

### 2.4 Platelet-rich fibrin

By centrifuging venous blood at various speeds, with or without thrombin and anticoagulants, to create a fibrin clot including platelets and white blood cells, autologous platelet concentrate (APC) is created (Mijiritsky et al., 2021). Biocompatible products containing cytokines, platelets, leukocytes and fibrin, which can be considered slow-release systems ensuring continuous delivery of active ingredients and growth factors for about 2 weeks (Ding et al., 2021), have now been used to treat scalp LP and genital sclerosing LP satisfactorily (Casabona et al., 2010; Bolanča et al., 2016). The first study on the application of first-generation APC platelet-rich plasma (PRP) in oral and maxillofacial surgery was published in 1997 by Whitman et al., but its extraction steps were cumbersome and could cause immune rejection or infection, so second-generation APC was born (Whitman et al., 1997; Choukroun and Miron, 2017). Second-generation APC platelet-rich fibrin (PRF), first identified in 2001, promotes vascular regeneration, regulates local immunity, and hastens wound healing (Bennardo et al., 2020). The PRF serves as a biodegradable scaffold for microangiogenesis and can direct epithelial cells to migrate to its surface (Dohan et al., 2006; Li et al., 2013). The benefits of PRF over PRP include, but are not limited to, the fact that it does not require anticoagulants, promotes faster wound healing, and boosts the immune system (Gunasekaran et al., 2021). In recent years, scholars have found PRF local injections to be comparable to tretinoin (TA) in relieving OLP pain, suggesting a role in EOLP pain control (Bennardo et al., 2021). However, one study found that TA showed better treatment results on the visual pain analog scale (VAS) and semi-quantitative REU (reticulo-erosion-ulcer) scores when both were injected at the same lesion site (Al-Hallak et al., 2023). Platelet-rich fibrin (i-PRF) and CS for EOLP both reduce pain and lesion size, but i-PRF is less expensive (Saglam et al., 2021). Patients resistant to topical corticosteroids may therefore profit from i-PRF.

### 2.5 Hyaluronic acid

Hyaluronic acid (HA) stimulates angiogenesis, moistens wounds, reduces exudation, possesses vasoprotective properties, and functions as fibrin in inflammatory and injured tissues to promote tissue regeneration. It is used in oral surgery and periodontal treatment. It was found that HA was comparable to 0.1% triamcinolone in reducing the VAS score and size of the lesions in OLP (Hashem et al., 2019). In a clinical study, effective control of OLP was observed in patients using an anti-inflammatory mouthwash containing 0.3% HA as well as topical tacrolimus, but the tacrolimus group showed better clinical performance at 3-month follow-up (Polizzi et al., 2023). The above studies suggest that HA may be effective in short-term localized applications, but its ability to play a role in long-term disease control is debatable.

### 2.6 Anti-metabolic drugs

Anti-metabolic drugs such as azathioprine act to inhibit cell proliferation by inhibiting the purine pathway and interfering with DNA synthesis (Broen and van Laar, 2020). Adverse effects of such drugs may lead to bone marrow toxicity and gastrointestinal and hepatic mucosal damage (Verma et al., 2001). In contrast, antimetabolites are rarely used clinically for the treatment of OLP (**Table 3**).

### 2.7 mTOR inhibitors

The mTOR signaling pathway regulates cell survival, proliferation, translation, autophagy, and cytoskeletal reorganization. It regulates cellular responses to dietary and oxygen status changes (Paghdal and Schwartz, 2007). Sirolimus binds to cell membrane proteins similarly to tacrolimus and was the first mTOR inhibitors (mTORIs) to receive approval from the US Food and Drug Administration (Yang et al., 2016). The IL-2 signaling pathway is blocked by sirolimus, which inhibits the mammalian target of rapamycin by directly interacting with the mammalian rapamycin complex 1 (mTORC1). This prevents the activation of T and B cells (Treister et al., 2021). Soria et al. treated seven patients with EOLP using topical Sirolimus; after three months, four patients were in complete remission and two were in partial remission. Although myelosuppression and hyperlipidemia are the most common adverse effects of oral sirolimus for the treatment of chronic refractory EOLP, sirolimus is not well absorbed into the bloodstream and side effects are rare (Soria et al., 2009) (**Table 3**).

### 2.8 JAK/STAT signaling pathway inhibitors

**Figure 2:**
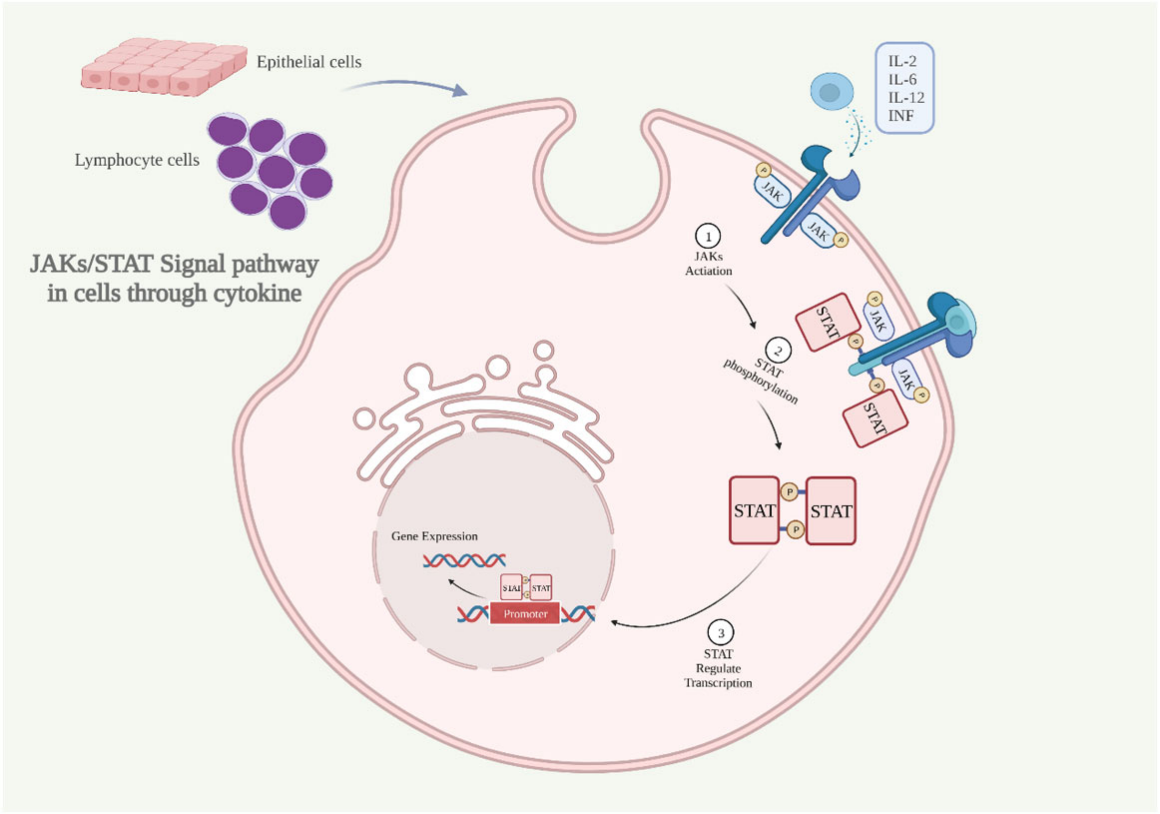
The JAKs/STAT signaling pathway is activated through cytokine **(A)** Inflammatory cytokines bind to the receptor, dimerizing it, activating JAKs, and phosphorylating the intracellular portion of the receptor. **(B)** STAT proteins bind at sites containing phosphorylated tyrosine. **(C)** STAT dimerizes and translocates into the nucleus to regulate gene expression. IL, interleukin; INF, interferon; JAKs and STAT, Janus kinases and Signal Transducers and Activators of Transcription (Created with BioRender.com).

Accidentally, Janus kinases and Signal Transducers and Activators of Transcription (JAKs and STATs) were discovered while studying the effects of cytokines such as interferon and interleukin (Stark and Darnell, 2012). JAKs are intracellular second messengers that are necessary for the cell to receive signals from extracellular cytokines (Schroder et al., 2004). JAK kinase has four isoforms (JAK1, JAK2, JAK3 and Tyk2), and STAT protein contains seven isoforms (STAT1, STAT2, STAT3, STAT4, STAT5A, STAT5B, and STAT6) (Nishio et al., 2001). JAKs and STATs are phosphorylated by type I and type II cytokine receptors, and then STATs enter the nucleus and regulate transcription as well as immune and blood processes (Leonard and O'Shea, 1998; Arbouzova and Zeidler, 2006; Yazdi et al., 2016; Sabat et al., 2019) (**Figure 2**).

It was discovered that JAK1 plays a crucial role in immunological and inflammatory responses and is engaged in IL-2 and IL-6 signaling (Migone et al., 1998; Haan et al., 2002). Abduelmula et al. found that MHCI expression was found to increase in keratinocytes, mainly through the JAK2/STAT1 pathway and inhibition of this pathway protects keratinocytes from cytotoxic responses (Abduelmula et al., 2023). JAK3, on the other hand, was found to be upregulated in peripheral blood Th1 and Th17 cells from OLP patients, accompanied by upregulation of STAT2 and STAT6 expression (Ociepa et al., 2022). Furthermore, IFN-γand CXCL10 were strongly expressed in lesions as well as in the serum of patients with LP, which was regulated by JAK1/2 (Flier et al., 2001). The aforementioned findings imply that the JAK/STAT pathway might be crucial in OLP.

The therapeutic effect of JAK inhibitors on OLP has been clinically proven. Tofacitinib inhibits JAK1/2/3 and, to a lesser extent, TYK2 (Hodge et al., 2016). Abrahim et al. found an overall effectiveness rate of 70% for tofacitinib in the treatment of LP by meta-analysis (Abduelmula et al., 2023). Baricitinib inhibited JAK1/2, and Moussa et al. discovered that a 63-year-old female patient with left buccal erosion improved after one month of oral baricitinib treatment, and that her oral irritation and distress were nearly eliminated after four months (Moussa et al., 2022). The adverse effects of tofacitinib and baricitinib seem to contain herpes zoster, occurrence of malignancy, and gastrointestinal perforation (Genovese et al., 2016; Strangfeld et al., 2017; Winthrop et al., 2017; Burmester et al., 2018). So far, they have not been found in the treatment of OLP. Upadacitinib is a JAK1 inhibitor but can also inhibit JAK2. Oral lesions in an EOLP patient receiving 15 mg of upadacitinib daily improved over time and experienced no side effects from the therapy. This outcome agrees with Neda’s observations (Kooybaran et al., 2021; Balestri et al., 2022). According to a recent case report from 2022, abrocitinib is a selective inhibitor of the JAK1 enzyme, patients with OLP who received a daily dose of 200 mg alone for 8 weeks and then 100mg for 4 weeks demonstrated full recovery of the right cheek lesion. The patient responded well to the medication, and there were no untoward occurrences (Gooderham et al., 2019; Solimani et al., 2023). However, adverse reactions have occurred in the context of treatment for atopic dermatitis (AD), generally at the respiratory and/or gastrointestinal level (Niculet et al., 2022). Peficitinib, a novel JAK3 inhibitor, was found to reduce enzyme activity and cell proliferation in an efficient manner (Qiu et al., 2019). One study reported that topical use of JAK3 enzyme inhibitors in cutaneous LP may have therapeutic promise (Samadi et al., 2017). Although tofacitinib, baricitinib, and upadacitinib all inhibit JAK2, there is no FDA-approval for the use of selective JAK2 inhibitor in OLP at the moment (Spinelli et al., 2021). It should be emphasized that it is not clear which isoform of the JAK enzyme is prominent in the pathogenesis of OLP, and more research is needed to develop more targeted drugs in the future (**Table 4**).

**Table 4:** JAK enzyme inhibitors in oral lichen planus.

Drug	Mechanism of Action	Indications	Method and Dose	Side Effect
Baricitinib	Inhibits JAK1/JAK2, blocks γc-chain, β-co-chain (IL-5, IL-130 and GM-CSF), gp6 (IL-12 family), interferon and interleukin (IL-23, IL-27, IL-19) signaling; inhibits allogeneic T cell responses to keratinocytes in vitro	Patients not responding to conventional treatment	Whole body: 3.4mg, ×2/d	Slight increase in cholesterol
Tofacitinib	Inhibition of JAK1/JAK2/JAK3 STAT1/STAT3/STAT5, competitively binds to the active region of adenosine triphosphate kinase and blocks activation of signal transduction	Application to patients with lichen planus of the scalp	Topical: 5mg in LP 2%, ×2/d Whole body: 10-15mg/d	Temporary abnormal hemoglobin and creatinine, mildly elevated triglyceride and cholesterol levels
Upadacitinib	Inhibition of JAK1 enzyme activity	Patients with erosive oral lichen planus	Whole body: 15mg daily	No significant adverse reactions
Peficitinib	Inhibition of JAK3 enzyme activity and JAK1/3-mediated cell proliferation	Applied to generalized lichen planus	Systemic dosing, exact dosage not yet known	No significant adverse reactions
Abrocitinib	Inhibition of JAK1 enzyme activity	Applied to generalized lichen planus	Whole body: 100mg/200mg daily	respiratory and/or gastrointestinal level

### 2.9 Antioxidant therapy

Some evidence suggests that the pathogenesis of OLP can be mediated by the oxidative stress (OS) state, which is also believed to facilitate the process of OLP’s malignant transformation (Amirchaghmaghi et al., 2016). The presence of reactive oxygen species (ROS) at the lesion increases the T lymphocyte-mediated inflammatory response, upregulates the expression of intercellular adhesion molecule (ICAM)-1 in keratin-forming cells, and disrupts their lipid membrane, resulting in increased T cell infiltration and ROS production (Gupta et al., 2017; Bao et al., 2022). Patients with OLP had higher levels of oxidative stress and lower levels of antioxidants, suggesting that oxidative stress contributes to the development of OLP (Wang et al., 2021). Many antioxidants have been used as adjuvant therapy for OLP, such as glutathione, coenzyme Q, lipoic acid, carotenoids, vitamins A, C, and E, and resveratrol (Thongprasom et al., 2011; Pisoschi and Pop, 2015). After 12 weeks of treatment, OLP patients who received topical selenium (Se) had substantially lower pain scores (NRS) than those who received corticosteroids, and the topical Se group experienced longer-lasting pain relief (Qataya et al., 2020). This suggests that Se alone may be helpful in the management of OLP with longer-lasting effects, improved pain alleviation, and no adverse effects.

### 2.10 Chinese herbal medicine treatment

Chinese medicine employs yin and yang, as well as the five elements, to restore internal organ function. Certain botanicals are immunomodulatory, anti-inflammatory, metabolism-promoting, and microcirculation-improving, according to modern pharmacology (Duan, 2015; Pang et al., 2018; You et al., 2020). Herbal medications with minimal adverse effects, such as Liu Wei Di Huang Wan and Tripterygium glycosides, are used to treat chronic oral diseases in China and other Asian countries (Ghahremanlo et al., 2019). Curcumin, a plant extract from turmeric with anti-inflammatory, antioxidant, and antitumor properties, has recently been studied in relation to OLP. Early OLP therapy with 6,000 mg per day of curcumin was effective (Chainani-Wu et al., 2012b; Chainani-Wu et al., 2012a). The use of curcumin and turmeric extracts to treat OLP, however, is disputed by certain researchers (Zeng et al., 2022).

### 2.11 Total glucosides of paeony

Total glucosides of paeony (TGP) is an active compound extracted from the root of Paeonia lactiflora that has immunomodulatory effects, and its components include paeoniflorin, hydroxypaeoniflorin, paeoniflorin, leuconidin, benzoylpaeoniflorin, etc. It is often used as an analgesic and anti-inflammatory drug for the treatment of diseases such as rheumatoid arthritis (RA) and skin ailment psoriasis (Jiang et al., 2020; Zhang and Wei, 2020). A double-blind, randomized, placebo-controlled study showed that TGP and Acitretin together improved the effectiveness of antipsoriasis treatment and decreased liver damage (Yu et al., 2017; Zheng et al., 2019).

Although TGP is used in the treatment of autoimmune diseases, its specific modulation of OLP is unknown (Jiang et al., 2020). TGP consistently reduces the number of Treg and Th1 and inhibits T-cell proliferation (Wang Y. et al., 2016). Wang et al. found that the NF-κB signaling pathway was significantly activated in OLP tissues, and TGP seems to inhibit the production of IL-6 and TNF-α in LPS-induced HaCaT cells in a dose-dependent manner, thus inhibiting the phosphorylation of IκBα and NF-κB p65 proteins (Yoke et al., 2006; Wang Y. et al., 2016). On the other hand, YAN et al. observed in a clinical study that peripheral blood IFN-γ and IL-10 levels were significantly elevated in patients with OLP after treatment with TGP, suggesting that TGP may play a role by increasing the expression of IFN-γ and IL-10 in peripheral blood (Yan and Z, 2016). These seem to reveal the tip of the iceberg of the mechanism of action of TGP for OLP treatment. The combination of TGP with triamcinolone acetonide and tacrolimus had a satisfactory therapeutic effect, and the percentage of fungal infections was not significantly different from the normal population (Marable et al., 2016). During TGP treatment, no liver or kidney damage or other side effects were observed, and a small number of patients experience diarrhea, which dissipates quickly (Li et al., 2014; Feng et al., 2019; Luo et al., 2019). The above findings suggest that TGP may be a strong candidate for the treatment of OLP (**Table 3**).

In terms of microbiology, it has been found that the combination of TGP and hydroxychloroquine can increase the growth of a variety of beneficial bacteria, inhibit the growth of dominant pathogenic bacteria, increase the diversity and abundance of intestinal microorganisms. Among them, the abundance of Lactobacillus, Bacteroides undulatus and Vibrio desulfuricans was significantly increased and the abundance of Bacteroides and alloprevotella was significantly decreased in the TGP+hydroxychloroquine group (Lu et al., 2020). However, the current study could not construct a complete intestinal ecosystem, and further studies are needed in the future to investigate the specific mechanisms of the microbial effects of TGP and hydroxychloroquine.

### 2.12 Micronutrients and EOLP

Micronutrients, such as vitamins and trace minerals, are crucial in determining one’s susceptibility to a number of systemic and oral disorders (Bhattacharya et al., 2016). A recent study reveals that OLP patients had lower levels of essential micronutrients for normal metabolism, such as iron, zinc, calcium, vitamin D, and vitamin B12, and higher levels of oxidants and homocysteine (Gholizadeh and Sheykhbahaei, 2021). Vitamin D inhibits B-lymphocyte differentiation and immunoglobulin secretion, and a growing number of studies have confirmed the link between vitamin D deficiency and OLP (Kujundzic et al., 2016; Zhao et al., 2019). The oral mucosa is exposed to environmental factors that compromise the immune system and increase tissue permeability due to epithelial thinning, mucosal inflammation, and atrophy from iron deficiency. Also, cell differentiation and proliferation require iron, which explains why supplemental iron decreases OLP lesions (Maggini et al., 2007). In particular, CD8^+^ and NK cells benefit from vitamin B12’s regulatory action as an immunomodulator of cellular immunity (Permoda-Osip et al., 2014). The combination of vitamin B12 and immunomodulatory drugs was effective in reducing autoantibody levels, pain, and lesion severity in patients with OLP (Chang et al., 2009; Lin et al., 2011).

### 2.13 Microbial agents

It has been reported that the oral cavity is home to more than 700 types of bacteria and 100 species of fungi (Escapa et al., 2018). Microorganisms are linked to the inflammatory cytokine production in OLP patients, including IL-1, IL-10, IL-17, and IFN-γ, the activation of T cells, and the elevation of protein expression linked to the response to oxidative stress (Jung and Jang, 2022). A variety of bacteria and fungi, including Lactobacillus, Bifidobacterium, and Saccharomyces species, also have distinct effects on the immune response. They control and modify the T regulatory/T helper 17 (Treg/Th17) axis, which shields the host from infections and reduces the overabundance of effector T cell responses (Ashraf and Shah, 2014; Yu et al., 2019). Accordingly, microbial therapy might make a great difference in the treatment of OLP.

Notably, the current microbiological therapies for OLP seem to be ineffective (Keller and Kragelund, 2018; Kragelund and Keller, 2019; Cosgarea et al., 2020; Marlina et al., 2022). Maukonen et al. were unable to detect any probiotics included in oral capsules in saliva samples (Maukonen et al., 2008). Svante et al. discovered that chewing gum containing L. reuteri lowered the levels of pro-inflammatory cytokines in gingival sulcus fluid (Twetman et al., 2009). Therefore, direct contact may be required for probiotics to colonize the oral mucosa. Nevertheless, other research indicates that probiotic colonization of oral biofilms seems to be a somewhat transient process (Yli-Knuuttila et al., 2006). Horz et al. discovered, for example, that Streptococcus salivarius K12 only colonized the mouth momentarily (Horz et al., 2007). After giving probiotic-containing dairy items to their patients for three days, Ravn et al. discovered that the probiotics had vanished from the surface of the teeth and were only slightly present in the saliva and oral mucosa (Ravn et al., 2012). The above results may be due to the inability of the current mode of delivery to act directly on the lesion site, and a probiotic delivery mechanism that operates directly on the lesion site might be created in the future, which would be a daring and interesting attempt.

## 3 Non-pharmacological treatment

### 3.1 Photodynamic therapy

Photodynamic therapy (PDT), a non-invasive, safe, and non-toxic treatment for oral precancerous lesions, has been shown to be beneficial (Ch, 2012). PDT consists of photosensitizers, light, and reactive oxygen species. Through biochemical interactions, singlet oxygen and free radicals from PDT induce cell death, membrane disruption, and protein inactivation at the lesion site (Hesse et al., 2020). According to Cosgarea et al., methylene blue-mediated photodynamic therapy (MB-PDT) decreased the number of CD4^+^ and CD8^+^ T cells in OLP-lesions and their capacity to produce IL-17 (Cosgarea et al., 2020). Mostafa et al. included 20 patients with EOLP and compared the effects of MB-PDT with topical CS and found that MB-PDT was effective in reducing pain and minimizing lesions (Mostafa et al., 2017). Jajarm et al. compared the efficacy of toluidine blue-mediated photodynamic therapy (TB-PDT) with topical corticosteroids in EOLP. During a one-month follow-up, no significant difference was found between the two, while the CS group seemed to be more effective in terms of pain relief (Jajarm et al., 2015). Chen et al. noted that saliva and soft tissue movements in the oral cavity result in incomplete photosensitizers absorption, and the more of the photosensitizer retained in the local lesion, the better the efficacy of PDT (He et al., 2020). Clinicians need to take these factors fully into account when selecting photosensitizers. Notably, Saleh et al. concluded that PDT is a more effective and safer treatment option for some patients with diabetes and hypertension (Saleh and Khashaba, 2022), and Sulewska et al. included 12 elderly patients with EOLP who showed significant healing or even complete remission of the lesions during a follow-up period of up to 12 months after treatment (Sulewska et al., 2017). The above results suggest that PDT offers a non-invasive treatment for oral mucosal lesions and may become an alternative and complementary method to those currently in use (**Table 5**).

**Table 5:** Non-pharmacological treatment oral lichen planus.

Treatment modality	Treatment Features	Applicable people
Surgical excision	Direct excision of the lesion	For recurrent lesions with a high risk of cancer
Laser treatment	Good tissue penetration, enhanced local blood flow, significant pain relief and accelerated healing of lesions	Medical treatment is ineffective and the lesion is extensive, making it unsuitable for surgery
Photobiomodulation therapy	Promotes cytochrome c oxidase (CcO) in the mitochondrial respiratory chain, driving adenosine triphosphate (ATP) production for pain relief, inflammation relief and tissue repair	
Photodynamic therapy	Produces ROS and free radicals to selectively kill inflammatory or malignant tissue without toxicity, low risk of complications, minimally invasive and minor side effects	
Psychological intervention	Therapeutic implications for people with depression and anxiety	Patients with depression, anxiety and other psychological disorders and bad moods with oral lichen planus
Inital periodontal treatment	Improves the oral microenvironment, creating conditions for reducing inflammation levels and promoting mucosal healing	Patients with oral lichen planus with periodontal disease

The effect of PDT on the oral microbiome has been poorly studied, and the available evidence suggests that PDT reduces clinical infections caused by drug-resistant Gram-positive and Gram-negative bacteria (Carpenter et al., 2015). For example, antimicrobial photodynamic therapy was applied to the treatment of peri-implantitis in a study by Dörtbudak et al (Magnet et al., 2001). This was also reported to be beneficial for the recovery of OLP patients treated with PDT to avoid complications due to postoperative infections (Cieplik et al., 2014; Kang et al., 2019). However, antimicrobial efficacy in the deeper layers of the oral biofilm remains controversial because bacteria embedded in the biofilm have a higher tolerance to antimicrobial agents (Mah and O'Toole, 2001; Marsh, 2004). Cosgarea et al. studied and analyzed OLP patients 28 days after PDT treatment, an analysis that included 18 oral microorganisms and found that the bacteria were not statistically different despite varying reductions in numbers (Cosgarea et al., 2020). More rigorous evidence for the effect of PDT on the oral microbiome is required, and that its antibacterial capacity is unlikely to be achieved by modifying the microbial makeup.

### 3.2 Photobiomodulation therapy

In 2014, low-level laser therapy evolved into photobiomodulation (PBM). PBM stimulates cytochrome c oxidase (CcO) in the mitochondrial respiratory chain, which generates adenosine triphosphate (ATP) and reduces tissue injury by momentarily elevating reactive oxygen species (ROS) in cellular organization (Kemper, 2018; Flores Luna et al., 2020). PBM is considered a novel approach to OLP treatment because of its ability to reduce pain, alleviate inflammation, and promote tissue repair in different pathological conditions (Nammour et al., 2021). Photobiomodulation is helpful at reducing pain and lesions in atrophic or erosive OLP, according to randomized, controlled, double-blind research, with no significant difference from topical steroid hormone use and showing good results at late follow-up (Ruiz Roca et al., 2022) (**Table 5**).

It is important to note that, like PDT, PBM is capable of causing some effects on microorganisms. Fukui et al. suggested that PBM irradiation may affect the metabolism and growth of Porphyromonas gingivalis, by the mechanism that visible and near-infrared light affects the bacterial cell cycle and growth mechanisms through major interactions with photosensitive molecules (Dadras et al., 2006). In addition, PBM may modulate the oral microbiome by improving salivary gland function, levels of interleukin-1 receptor antagonist, interleukin-10, and stimulate the immune system (Ailioaie and Litscher, 2021).

### 3.3 Laser treatment

Laser irradiation has been postulated to have an anti-inflammatory effect. It decreases the chemotaxis of polymorphic nuclei. and has a thermal effect, leading to microbial cell wall degradation, protein denaturation, and ultimately fungal cell death (Agha-Hosseini et al., 2012; Momeni et al., 2022). Potentially cancerous conditions are treated with CO_2_ laser technology (Flynn et al., 1988; Humphreys et al., 1998; Dong et al., 2019). The CO_2_ laser’s thermal impact carbonizes and vaporizes tissue, closing blood arteries and lymphatic vessels, destroying nerve endings, and sterilizing incisions.

The main laser treatments are neodymium-doped yttrium aluminum garnet (Nd : YAG) and Erbium-doped yttrium aluminum garnet (Er : YAG) laser (ERL) irradiation (de Pauli Paglioni et al., 2020). The Nd : YAG laser, a 1064 nm infrared laser, penetrates deeply into tissues, increases local blood flow, reduces pain quickly, and speeds up recovery (Kolli et al., 2021) (**Table 5**). Khater et al. treated EOLP patients with Nd : YAG laser three times a week for one month and found that it greatly reduced clinical symptoms without major side effects (Khater and Khattab, 2020). After irradiating Candida albicans and Streptococcus pyogenes strains *in vitro* using a Nd : YAG laser, Grezch-Lesniak discovered that Candida albicans and Streptococcus pyogenes counts declined and a substantial fall in bacterial metabolic levels was also observed (Grzech-Leśniak et al., 2019). As a result, in addition to its therapeutic purpose, the Nd : YAG laser also has potential for fungal control following topical hormone treatment in patients with OLP. Er : YAG-based high-intensity laser therapy (HILT) is a safe and effective method for removing OLP tissue (Fornaini et al., 2012; Broccoletti et al., 2015; Tarasenko et al., 2021). Er : YAG therapy in conjunction with 0.5% H_2_O_2_, 0.5% NaOCl, or 0.03% chlorhexidine effectively decreased the oral flora of Streptococcus gordonii, Clostridium nucleatum, and Porphyromonas gingivalis (Golob Deeb et al., 2019). The observation that the oral detection rate of P. gingivalis in OLP patients was substantially greater than in the healthy population (Ertugrul et al., 2013), this may imply that the mechanism of action of Er : YAG laser in the therapy of OLP involves a reduction in the microbial load.

### 3.4 Surgical excision

In general, EOLP lesions are difficult to treat and frequently recur. Surgical treatment may be an option for long-term, untreated lesions due to the increased risk of malignancy (Tarasenko et al., 2021). In a case report, the decision to excise the right buccal mucosal lesion was made after the use of local steroids, systemic steroids, hydroxychloroquine, and even intralesional steroids. Surprisingly, after 2 years of follow-up, recurrence was not detected (Hadiuzzaman et al., 2013). In addition, a subset of physicians have attempted to treat refractory EOLP with free palatal mucosa grafts (Kim et al., 2018). However, the efficacy and treatment criteria for this treatment modality have not been clearly established (Yan et al., 2017). Surgical excision looks to be more complete than conservative treatments and reduce lesion recurrence, although recurrence rate comparisons require larger samples and longer-term follow-up. Second, surgery is more costly, has limited therapeutic value, and is only suitable for those with limited and persistent erosions and lesions in risk areas with some cancer risk (**Table 5**).

### 3.5 Psychological intervention

Modern culture and medicine have made psychological factors more critical in many conditions including OLP (Hampf et al., 1987). There is growing evidence that mood disturbances is an important factor in the onset and amplification of OLP, especially in patients suffering from depression, anxiety, and acute stress (Song et al., 2021). Paranoia, anxiety, and depression were more pronounced in the reticular and erosive OLP group compared to the control group (Ivanovski et al., 2005). Depressive symptoms were seen in 41.66% of OLP patients and 22.91% of control patients in a clinical observation involving 48 people (Alves et al., 2015). In addition, patients with more severe lesions tend to have more pronounced anxiety (Zucoloto et al., 2019). Gabriella et al. found a significant effect of different personality types on the outcome of OLP patients, suggesting that psychological factors of personality play an integral role in OLP pathogenesis (Gabriella et al., 2021). Another study used the GAD-7 and PHQ-9 to compare moods in OLP patients and healthy people. The GAD-7 and PHQ-9 scores of OLP patients and controls differed significantly, suggesting that mental illnesses may be linked to OLP (Wiriyakijja et al., 2020). The aforementioned evidence suggests that doctors should pay attention to psychological issues in OLP patients to increase effectiveness and tailor pharmacological and psychological treatment approaches (**Table 5**).

### 3.6 Initial periodontal treatment

Chronic periodontitis (CP), the most common reason for adult tooth loss, is a chronic inflammatory disease that is primarily caused by bacteria and driven by the host immune system (Rai et al., 2016). Significant risk factors for OLP progression include dietary habits, tension, and poor oral hygiene; therefore, it is necessary to investigate the relationship between periodontal health and OLP (Xue et al., 2005). In previous studies, there was a complex interaction between OLP and CP. Hu et al. used the RAE scoring system to evaluate intraoral lesions in OLP patients with CP and the average gingival index (GI-Avg) to evaluate primary gingival inflammation and found a mutually reinforcing effect between OLP and CP (Wu et al., 2022). ZHAO et al. discovered that patients with EOLP exhibited significant improvement in OLP lesions and symptoms following one month of periodontal treatment, indicating that periodontal treatment is clinically significant for EOLP (Zhao, 2018). A meta-analysis revealed that the clinical indicators of bleeding on probing and probing depth were substantially linked with OLP, which may be owing to the patient’s incapacity to perform appropriate oral hygiene maintenance due to the uncomfortable sensation at the site of the lesion (Sanadi et al., 2023). The initial periodontal treatment in treatment of EOLP is promising. When treating OLP, particularly EOLP, clinicians should also take the patient’s periodontal health into account (**Table 5**).

Besides, smoking, diabetes, and obesity are among the many frequent systemic risk factors for periodontal disease and smoking appears to be able to interrelate with OLP alone, in addition to affecting OLP through periodontitis (Genco and Borgnakke, 2013). Smoking enhances the expression of TLR-34 and CD34 in OLP lesions, which can promote an inflammatory response. It may also lead to increased microvascularization in OLP lesions, which exacerbates cancerous tendencies (Kłosek et al., 2011; Amin et al., 2020). Furthermore, Alrashdan et al. discovered that smoking lowers macrophage expression in OLL, which may change immune surveillance and malignant transformation pathways (Alrashdan et al., 2016a). A meta-analysis of OLP patient data revealed that smokers had a significantly greater rate of malignant transformation than nonsmokers (Aghbari et al., 2017). It is suggested that we should not only focus on the patient’s disease state but also monitor and manage the series of behaviors of clinical patients that are not conducive to disease recovery.

## 4 Summary and outlook

OLP is a chronic inflammatory disease characterized by relapses and delayed healing, and thus has a prolonged detrimental effect on patients` quality of life. For asymptomatic reticular OLP, routine observation and testing are usually adequate. Low, brief doses of systemic steroids taken orally may be used to treat acute exacerbations or persistent, considerably deteriorating OLP lesions, but prolonged use is to be avoided. Topical calcium phosphatase inhibitors are the second most affordable and efficient form of therapy after topical corticosteroids, according to a comparison of the financial benefits of various therapeutic approaches. PDT sessions are the most expensive, despite evidence that they are more effective and have fewer side effects than other treatment methods (Sandhu et al., 2022). Although JAK inhibitors are less commonly used in OLP, there is a potential that they may be effective therapeutic agents for OLP, especially when conventional drugs are ineffective. If conservative treatments are not effective, laser and surgical treatments are also available, depending on the lesions and the patient’s wishes.

Most of the evidence regarding the involvement of microorganisms in the pathogenesis of OLP has not been conclusively demonstrated. More studies are required to unveil the interactions between the oral microbiome (bacteria, host metabolites, and microecological environment) and OLP (Berg et al., 2020). In addition to this, there is a need to explore the efficacy of oral probiotics as well as the mode of administration, which usually requires more and longer-term research observations.

The main goal of clinical OLP treatment is to lessen the severity of local inflammation while reducing disease recurrence and preventing malignant transformation. Owing to the risk of malignancy in EOLP, timely treatment plan adjustments, frequent medical follow-up, and a biopsy if needed are required. In addition to conventional medications and physical therapy, treatment strategies should also take into account the patient’s psychological characteristics, oral hygiene, systemic underlying diseases, smoking and alcohol abuse, and other adverse habits to obtain a more favorable therapeutic outcome (**Table 5**). Overall, a multi-faceted, multi-dimensional approach to the treatment of EOLP may be the only solution that can truly reduce disease recurrence and even restore immune homeostasis and cure the disease.

## Author contributions

TW: Investigation, Project administration, Writing – original draft, Writing – review & editing. YB: Investigation, Writing – original draft, Writing – review & editing. YJ: Investigation, Writing – review & editing. FC: Conceptualization, Funding acquisition, Project administration, Resources, Supervision, Writing – review & editing.
